# Patient and public involvement in doctoral research: reflections and experiences of the PPI contributors and researcher

**DOI:** 10.1186/s40900-020-00201-w

**Published:** 2020-05-11

**Authors:** Shoba Dawson, Angela Ruddock, Veena Parmar, Rebecca Morris, Sudeh Cheraghi-Sohi, Sally Giles, Stephen Campbell

**Affiliations:** 1 Centre for Academic Primary Care, Population Health Sciences, Bristol Medical School, University of Bristol, Bristol, UK; 2 NIHR Greater Manchester Patient Safety Translational Research Centre, Centre for Primary Care, Division of Population Health, HSR & Primary Care, School of Health Sciences, Faculty of Biology, Medicine and Health, University of Manchester, Manchester, UK

**Keywords:** Patient and public involvement, Doctoral research, Reflections, Impact

## Abstract

**Plain English summary:**

There is evidence in the literature showing that involving patients and the public in health research can have a positive influence on quality, relevance and impact of research. However, patients and the public are not always involved in all stages of the research. There is often no explanation as to why they were only involved in some stages of the research and not others. Additionally, there is often no description of researchers’ or PPI contributor’s experiences of involvement. This also raises another issue which is a lack of recording of impact such involvement can have on the research process and the people involved in the research. In this paper, we present what PPI in a doctoral research should look like by providing a detailed description of how involvement occurred from pre-funding to dissemination stages of the research process. We provide some practical examples of how this was done and how involving patients made a difference to the research project. Finally, we present reflections from the patient and public contributors and the researcher on involvement in this project along with some recommendations for future doctoral and postdoctoral researchers considering involving public/patient contributors in their research.

**Abstract:**

**Background**

Patient and Public Involvement (PPI) has received considerable attention in the last two decades and working in partnership and co-design have now become a prerequisite in health services research in the UK. However, there is a lack of evidence and consistency in recording PPI and related activities. Researchers and PPI contributors are encouraged to record and reflect on the impact of PPI on research. There is significant variation in the way PPI contributors are involved, and it is often limited to some stages of the research cycle than others, without any reflections on the decision-making process for such involvement or any transferable learning. This has resulted in failure to provide a narrative of the research journey including researchers’ and PPI contributors’ personal reflections of involvement. Therefore, this paper provides an exemplar of what PPI in a doctoral research context should look like by providing a detailed account of how PPI was embedded in a doctoral research project, the PPI contributors and researcher’s reflections and key recommendations for involving people specifically in doctoral research.

**Methods**

A reflective approach was taken using data from PPI contributor and researcher notes, e-mail correspondence, meeting notes. Data is presented narratively to reflect on the experiences of involvement throughout the research cycle.

**Results**

Undertaking PPI enhanced the quality and relevance of the doctoral research, contributed to the recruitment of study participants, data analysis and dissemination. Building trust and relationships with PPI contributors was key to continued involvement throughout the life of the project and beyond. There is a need to adopt flexible approaches rather than a one-size-fits-all model when working with PPI contributors. Reflections by PPI contributors and the researcher emphasises that involvement was a rewarding experience.

**Conclusions**

This paper contributes to the wider literature by providing an exemplar of how PPI can be embedded in doctoral research and demonstrates the value of PPI to the research process and the individuals involved. We also present recommendations on how PPI can be incorporated by doctoral and postdoctoral researchers when planning PPI in their research project.

## Background

Patient and Public Involvement (PPI) has received considerable attention both in the UK and internationally in the form of other comparable initiatives such as participatory approaches and public engagement. Internationally, institutions such as INVOLVE (UK), Patient-Centered Outcomes Research Institute (USA) and Strategy for Patient-Oriented Research (Canada) have been established to fund and promote PPI in health services research [1–3].

INVOLVE defines public involvement as “research carried out ‘with’ or ‘by’ members of the public rather than ‘to’, ‘for’ or ‘about’ them [4]. The rationale for PPI in health research is deep-rooted in the claims that PPI can have an impact on research quality, relevance, impact and integrity [5–7]. Three main reasons for PPI in health services research include normative or emancipatory, consequentialist or efficiency-oriented and political and practical. Normative or emancipatory reasons assert that patients have a right to be involved in research that might affect them and reduce power imbalances between researchers and PPI contributors [8–10]. The basis of consequentialist or efficiency-oriented reasons [9, 11] are that bringing a lived experience and real-world perspective contributes to improving the efficiency and value of research through various mechanisms [12]. Lastly, the rationale of the political or practical grounds suggest that spaces that offer co-construction of knowledge through alliances between researchers and patients can increase the accountability and transparency of research [12–14].

INVOLVE’s definition of public involvement embraces aspects of all three sets of reasons mentioned above. PPI is now well embedded that it is a funding requirement by the National Institute for Health Research (NIHR) and other funders for applicants to provide information on how PPI has and will continue to inform the proposed research [15]. Existing literature suggests that involvement happens at different levels (i.e. consultation, collaboration or user-led) and different stages of the research process. There are numerous examples of the impact of PPI such as securing funding, designing study protocols, choosing relevant outcomes and success in achieving recruitment targets of participants [16, 17]. However, there is considerable variation in the extent of, and approaches to, involvement and who is involved. Studies generally do not offer any rationale for researchers’ decision to involve PPI contributors in some stages of the research process over the others or a lack of continuous involvement, highlighting the gap between intended and actual involvement in practice [18–20].

While there is evidence in the existing empirical literature around the experience of PPI contributors and/or researchers on involvement and its impact, there is limited evidence emphasising the involvement process itself. For example, there is a little evidence discussing researchers’ experience of conducting the research including a description of where the researchers started, who influenced the different decisions, what they learned from these conversations, what changed as a result [21], researchers, or PPI contributors’ reflections. Therefore, this paper aimed to address a gap in the literature by providing an exemplar that focuses on the journey of PPI within this doctoral research as it concentrates on the following aspects: firstly, how PPI contributors were involved throughout the research cycle. Secondly, it presents the personal accounts of PPI contributors as well as the researcher reflecting on their experiences of involvement. Lastly, we highlight some recommendations to facilitate long term involvement from our experience.

### The research study

The examples, reflections and recommendations described in this paper are based on a doctoral research project funded by the NIHR Greater Manchester Patient Safety Translational Research Centre (Greater Manchester PSTRC). The study explored the views and experiences of people of South Asian origin on PPI and health services researchers’ experiences of involving people from Black, Asian and Minority Ethnic (BAME) groups in research. Ethics approval was granted in May 2015 by The University of Manchester Research Ethics Committee. Fifty-four participants were recruited with one-to-one semi-structured qualitative interviews undertaken by SD. The main findings from this study will be covered in forthcoming publications.

## Methods

This paper provides a descriptive account of PPI process, personal reflections of both the PPI contributors and the researcher on their experience of involvement in this research and its impact on the involvement process. The reflections presented in this paper are based on retrospectively examining the e-mail correspondence, documents with track-changes and notes from meetings. They are based on overall experience at the end of the project.

## Results

### Who was involved?

**Fig. 1:**
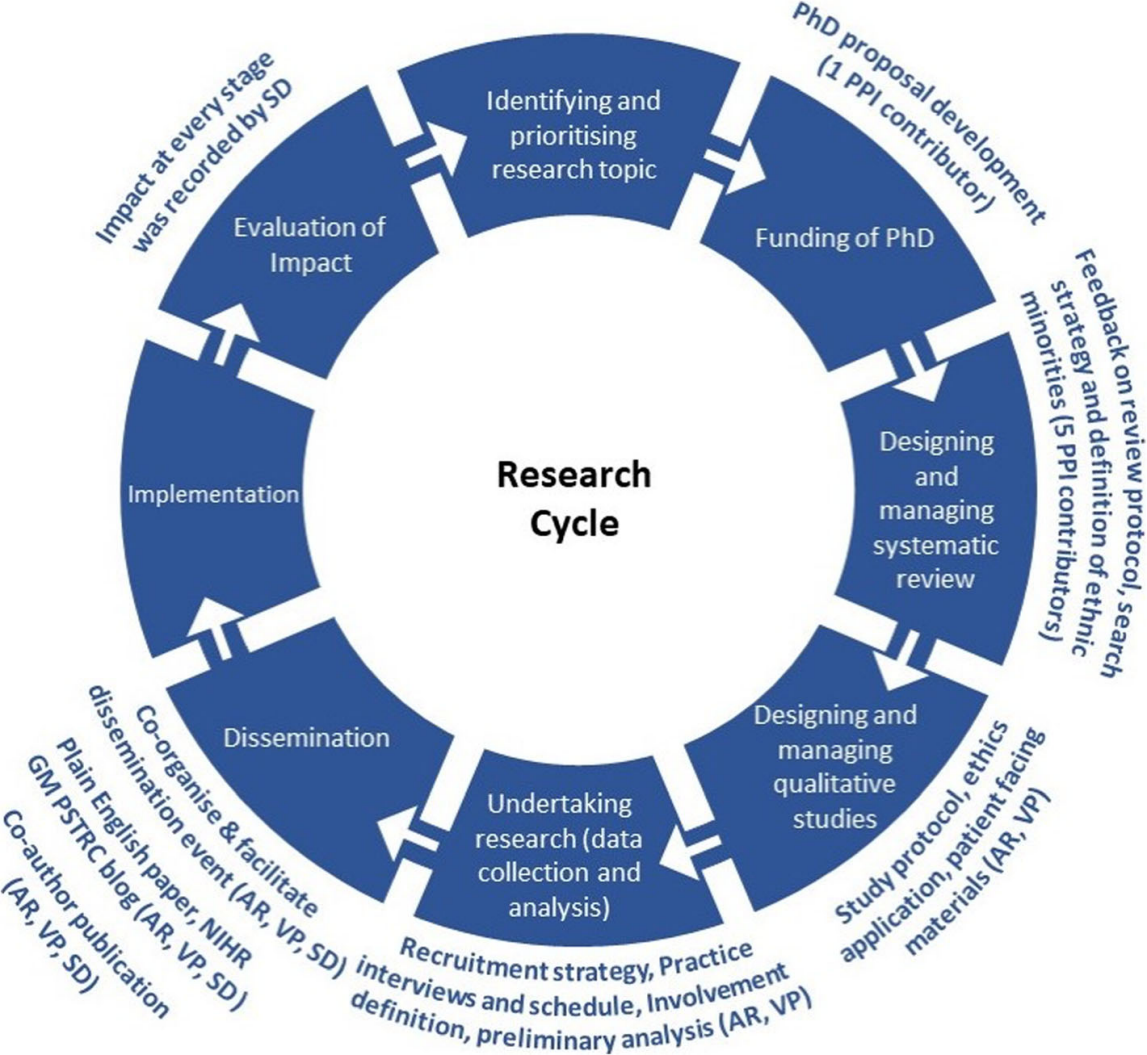
PhD Research cycle and involvement at different stages

Generally, researchers recruit PPI contributors to their study either because of PPI contributors’ experience of a health problem (e.g. living with a particular condition, carer of someone with a health problem), or using particular health services or treatments. Typically (more often than not), these PPI contributors also have other forms of experiential knowledge through sources such as experience of involvement in research which may or may not be factored in by the researchers when considering *whom to involve* in their study. Given that the current doctoral research was about PPI focusing on inclusivity and diversity, efforts were made to primarily recruit PPI contributors from BAME backgrounds and individuals with and without prior PPI experience post-funding. Additional PPI contributors with extensive PPI experience were recruited and involved solely in the systematic review. Using different approaches allowed consideration for whether PPI contributors’ expertise were appropriate for the involvement purposes. Table 1 provides a breakdown of how intended involvement translated into actual involvement in practice at different stages of the research project. It also illustrates training offered to the PPI contributors and impact at different stages of the research cycle (Fig. 1).

**Table 1:** Intended and Actual involvement opportunities in doctoral research

Timescale	Intended Involvement in research activities	Actual involvement in research activities	Training provided by SD	Impact
Short-term (10/2013–06/2014)	- Involvement in preparing PhD proposal for funding - Reviewing the proposal and providing feedback - Involvement in systematic review o Reviewing and commenting on the review protocol o Further stages of the systematic review	- Involvement in preparing PhD proposal for funding - Involvement in systematic review o Reviewing and commenting on review protocol	- Educational session on what a systematic review is and the process involved when undertaking a review.	- Validated the need to explore the topic area and develop research question. - Additional terms to search strategy, definition of BAME groups when exploring from an international perspective
Medium-term (07/2014–12/2015)	- Reviewing and commenting on: o Study protocol o Ethics application form, especially those sections that need to be writing in lay language/lay summary o Participant information sheets and consent forms o Topic guide for interviews	- Reviewing and commenting on: o Study protocol o Ethics application form, especially those sections that need to be written in lay language/lay summary o Participant information sheets and consent forms o Topic guide for interviews o Practice interview	- None	- Ensured information sheet and consent form was in plain English and jargon free - Changes to wording of topic guide and order of questions, re-define public involvement - Practice interviews increased researcher confidence
Long-term (05/2015–09/2017)	o Participant recruitment strategies o Data analysis o Dissemination of research findings (co-organising and facilitating the dissemination event). o Co-authoring documents, for example: summaries of research findings, publications	o Participant recruitment strategies o Data analysis o Dissemination of research findings through co-facilitation of the event o Lay summary part of doctoral thesis o Co-author reflective paper	- Educational session on what a thematic analysis is and the processes involved	- Enabled swift recruitment of study participants - Discussing findings helped identify other topics that required further exploration - Co-facilitation of dissemination event ensured engagement of all participants and co-authoring papers to share experiences from different perspectives.

#### Pre-funding

When funding opportunity for the PhD was advertised, the proposal was written with advice sought from a PPI contributor who was recommended by the co-author (SG). During the initial phase, the ideas for this research proposal were discussed, and feedback was sought on the research proposal. This PPI contributor suggested that efforts should be made to increase the diversity of PPI contributors involved in this study; therefore, their involvement was limited to the pre-funding stage.

#### Post-funding- recruitment of PPI contributors

Once funding was received, two new PPI contributors (XY and AR), who were part of the Greater Manchester PSTRC Research User Group (RUG) were recruited to be involved in the PhD research project. Both PPI contributors were from BAME backgrounds and were interested in improving care, access and support for BAME groups. The first PPI contributor (XY) was involved with various groups and forums but had no experience of PPI in a research setting. The second PPI contributor (AR) is from a BAME background and was involved in different PPI groups, with a keen interest in making PPI in health services research more inclusive, i.e. widening the types of people who become involved. The former (XY) PPI contributor was only involved in reviewing the systematic review protocol (see Table 1) as they left the Greater Manchester PSTRC RUG due to family commitments during the second year of the doctoral training. A colleague helped identify a new PPI contributor (VP) to replace this individual; VP had no prior experience of PPI. She is from a BAME background with interest in the health of BAME groups and keen to help by getting involved in this research study. Both AR and VP were involved throughout the project with VP being involved post-systematic review.

### Initial PPI meeting and practical considerations

We arranged an initial face-to-face meeting with the two PPI contributors (AR and VP) individually (between September 2013 and February 2014). We presented the study aims, expectations of the doctoral researcher and supervisory team, PPI contributors’ expectations regarding their involvement, preferred methods and frequency of communication and meetings, and terms of reference including ground rules (based on the Greater Manchester PSTRC RUG terms of reference).

Researchers’ expectations regarding PPI involvement in doctoral research were presented in the form of a pre-determined list of potential involvement opportunities. These were developed by the doctoral researcher and the supervisory team to involve the PPI contributors throughout the research process (see Table 1). SD discussed potential involvement opportunities, including explaining the different components of the PhD and the research process to the PPI contributors. They had the opportunity to be involved in all or any of the research processes presented below, allowing for flexibility. Accessibility of the venue for meetings was also discussed. As the PPI contributors were happy to meet at The University of Manchester, efforts were made to hold meetings in a different location that was more accessible within the campus. Therefore, meetings were held in different locations within the University campus depending on availability of rooms.

All meetings (*n* = 16) were planned 3–4 weeks in advance and were held on a day and time that was convenient for the PPI contributors during 4 years at a University location. Except for the initial meeting, where one of the supervisors (SG) was present to support the researcher (SD), all the other meetings involved only the researcher and the PPI contributors. Additionally, contact was maintained in-between via e-mails and/or telephone conversations on a regular (once a month/every other month) basis to provide a progress update and seek input. All documents that required input from the contributors were sent primarily via e-mail, and where the document was lengthy, a hard copy was also posted.

PPI contributors were reimbursed for their time and fees (£20.00 per hour) for face-to-face meetings, in line with the Greater Manchester PSTRC payment policy, which adheres to NIHR INVOLVE recommendations.^1^ PPI contributors were also reimbursed for travel and any related costs (i.e. carer allowance, childcare). Training needs were considered, and it was agreed that PPI contributors would receive training by the doctoral researcher where appropriate and possible. Educational sessions on different topics such as systematic reviews, qualitative research and facilitation were provided by SD. During the duration of the project, SD also had the opportunity to attend a variety of PPI training offered by Greater Manchester PSTRC, locally and nationally by other organisations. This included introduction to PPI, PPI in evidence synthesis, principles and applications of PPI, sharing practice and innovation for PPI in research.

### Involvement in the systematic review

Three other PPI contributors in addition to AR and XY were involved in reviewing the systematic review protocol. These three PPI contributors had considerable experience of PPI, and two of the three had some experience of being involved in systematic reviews. The decision to involve additional PPI contributors at this stage was to allow the opportunity to embed PPI in the development of the systematic review protocol. Involving these three PPI contributors led to adding two new terms (steering group and patient advocate) to the search strategy and consider how minority ethnic groups were defined in the literature [22].

### Involvement in other stages of the doctoral research project

Except for the systematic review, where the PPI’s contributors’ involvement was limited to reviewing the protocol [22], both AR and VP were involved in all stages of the research process (see Fig. 1) including contributing to the lay summary, which formed part of the doctoral thesis.

The research stages involved the development of the study protocol, University ethics application form, study materials, data management and analyses and research article writing. Study materials included information sheets, consent forms, topic guides for interviews and advertisement to recruit participants. The researcher (SD) provided AR and VP with a short educational session on processes and steps involved in data management and analysing qualitative data. A few (*n* = 6) anonymised transcripts from the interviews with South Asian participants (*n* = 27), and interviews with researchers (n = 27) were shared to help PPI contributors gain a better understanding around processes involved in analysing the data and also to elicit their views regarding the themes that emerged from the data. Furthermore, preliminary findings were also presented to gain feedback regarding any other topics that might require further consideration when interviewing study participants.

Finally, a ½ a day dissemination event was held at the Manchester Museum on The University of Manchester campus in October 2016. When participants consented to take part in the qualitative studies, they had the option to provide their contact details if they wanted a copy of the study findings. Study participants (South Asian patients/members of the public and researchers) were invited to attend this event. Twenty-two of the Fifty-four participants who participated in the interview study as a part of the wider doctoral research attended this event with the majority being lay participants (*n* = 19) and 3 being researchers. This event was organised in collaboration with AR and VP, who helped plan the event and, on the day, facilitated discussions with study participants. While the purpose of this event was to share the study findings, the researcher also used this as an opportunity to do member checking, i.e. taking findings back to the study participants for their confirmation that the researcher’s interpretation of the data accurately reflects participants’ intended meaning [23].

SD met with AR and VP before this event to discuss the processes involved in facilitating this meeting and offer any additional support, they might require on the day along with an opportunity for them to clarify any concerns they might have. Based on their suggestion to accommodate cultural considerations for the South Asian participants (female, Pakistan origin), a female PhD student proficient in Hindi/Urdu and three other PhD students were recruited, and support was provided to enable facilitation of discussions with the participants.

### PPI contributors’ reflections of being involved

#### Angela Ruddock- public contributor

I was born in London and moved to Manchester in 1984, following completion of university degree in Microbiology, and worked in education administration and then in Human Resources within the higher education sector. Then I joined the NHS, when after 35 years’ service, I retired in 2009. I became interested in clinical research following the pursuit of a personal interest in diabetes, which is prevalent in my family and after following the progress of the treatment of 2 close relatives suffering from Diabetes and late on dementia.

I became a member of the Salford University Hospital NHS Foundation Trust and joined a PPI group of the North West Diabetes Research Group based in Salford in 2010. I then became aware of the PRIMER group (Primary care Research In Manchester Engagement Resource) based in the Centre for Primary Care at The University of Manchester and funded originally by the NIHR School for Primary Care Research. This forum was particularly attractive to me because of the wider aspects covered within it, not just diabetes but also other chronic illnesses affecting the elderly and other often vulnerable groups. I have since been involved, in a number of lay public groups, by providing feedback and commentary on a number of research projects. One of the events I attended during this time was a *Hack Day* where researchers meet up with members of the public and pull together ideas for research projects. When I joined these groups from 2010, I was particularly interested in the fact that there were very few, if any, public contributors from black and minority ethnic (BAME) communities. Very often I was the only such member at any of the meetings.

I was introduced to SD, who at that stage had little research experience, at this Hack event, and I discovered that she was doing a research project on this very subject. I was very pleased to be invited to be involved in her PhD project. SD began setting-up a PPI group comprising of myself and 2 other public contributors, one experienced in public involvement in several NHS projects and who had particular carer responsibilities and therefore experience, and one with no previous experience of PPI, although with lived experience as a parent and partner with a family. Shoba made a point of referring to us from a patient/public perspective as well as liaising with researchers (her supervisory team) experienced in involving members of the public.

Shoba shared with us from the outset her aims and objectives, including the definitions of public involvement and engagement, literature existing at that time. There was very little, if any, research relating to the lack of BAME public contributors. We provided input, by comments and our own experiences at different stages of the research process which I feel made Shoba re-appraise approaches to advertising participant opportunities, and which mediums could be used for participant recruitment.

We trialed practice interviews with Shoba so that she was able to use Plain English which would be clearly and easily understood (see Table 1). We also suggested how interpreters could be used particularly when targeting older women from South Asian communities. We felt it was important for Shoba to feel confident while interviewing and consider how best to explain the concept of PPI to lay participants, as most of them would not be familiar with it. We also commented on ways people could be attracted to attend, for instance through community associations and venues where particularly women will feel comfortable meeting and confident in being able to discuss personal issues.

Shoba then met with us to discuss some of the anonymised interviews and the initial findings, which we found not only interesting but, in many cases, resonated with our own personal experiences. It was fascinating that our responses could be identified as common issues for different minority groups, including eastern European, and groups marginalised through mental health issues, homelessness and socio-economic factors.

Following discussions, we suggested that Shoba compile a Plain English paper to present to the PRIMER Group based in the Centre for Primary Care, The University of Manchester. This session and discussions with them also enabled Shoba to validate the findings to some extent and think about other topic areas that required further attention. Both I and one other public contributor Veena also supported Shoba by acting as facilitators for the dissemination event at the end of the data analysis phase, where both lay and researcher participants were invited to attend this 1/2 a day event. The study findings were discussed to obtain wider views; this helped identify if the findings resonated with the views of participants as well as reflected on the recommendations.

This was a 4-year project, and it made me appreciate how long these studies take. We were fortunate that Shoba ensured that we were regularly updated and informed of progress, so we did not feel that we were out of the loop. One final point to add is that care was taken to ensure that we were compensated for our time in reviewing the documentation as well as the physical arrangements for meetings. We were also advised of the progress leading to the award of the doctorate this year.

#### Veena Parmar- public contributor

I am a second-generation Indian-born in Nairobi and educated in the British colonial system. My family is a blend of Anglo-Indian Portuguese culture. It’s great when we have family gatherings to exchange our various views. Since being married, I have lived in and around Manchester. I had only heard about people who were participating in clinical trials. I was persuaded to get involved by Shoba in her research. I was reluctant at first, but Shoba really encouraged me to come and meet her and the other PPI partner. At the meeting I found the topic interesting and agreed to continue my involvement. Between Angela and myself gave pointers to Shoba about how to advertise and recruit people via Asian shops, ethnic centres and religious meeting places. Notices were put in various public places for ethnic minorities (Table 1). I also offered suggestions about how to work through community leaders and how to approach women in socially conservative communities.

She then met with the community group gatekeeper to ask for permission to chat about her research to the community centre users i.e. South Asian participants. With the gatekeepers’ permission, it was possible to speak to people during their group activity sessions. I also took part in a mock interview to test the interview questions before Shoba recruited study participants. She asked for our feedback and it was then incorporated, this process also helped Shoba to gain confidence in interviewing study participants. There were lots of obstacles to overcome the cultural boundaries. For example, there were lots of shy women who were also worried about anonymity and confidentiality of information shared, therefore did not want Shoba to record their interviews. In such situations, she explained the process of anonymity and confidentiality and its implications and took notes instead to enable their participation and to make them feel comfortable and gain their confidence. Their interviews were done with utmost sensitivity.

I found this study research that Shoba carried out very interesting. Shoba took us through the process of analysing interview data, and we read some anonymous transcripts and notes and discussed the findings in relation to our experiences as well. I enjoyed reading case studies, facilitating the event and the whole process; we had dynamic meetings where we bounced off ideas and we often overstayed our meetings. I would recommend anyone to get involved in research. My advice to anyone who wishes to get involved would be to ensure that you understand what you are getting yourself in to and ask questions. Keep an open mind and you will learn a lot.

### Researcher’s reflections on involving PPI contributors and impact

While existing literature suggests that PPI can often be perceived as a time-consuming exercise and resource-intensive, planning and involving PPI contributors from the outset enabled the researcher to plan a budget for PPI covering the research cycle. Building and maintaining trust and relationships were crucial to have sustained ongoing involvement and developing a positive working relationship. Understanding PPI contributors’ motivations for involvement and their expectations helped manage expectations from the outset as they enabled the development of a close partnership between the researcher and the PPI contributors. The researcher (SD) planned involvement in this doctoral research from the outset. When planning PPI for this research project, discussions with supervisors and co-authors led to an agreement that involvement should be less categorical (e.g. consultation, collaboration) and more flexible approaches should be adapted. For example, SD intended to involve the PPI contributors throughout the systematic review phase. Still, in practice, involvement was limited to the development of the protocol as PPI contributors did not feel that they had the skills to be involved further in the review. Therefore, involvement in this project was fluid and enabled flexibility throughout the research process with PPI contributors having the option to choose the extent of involvement and be involved in a way that was meaningful for them as reflected by the two PPI contributors (AR and VP). Several factors contributed to the decision-making regarding the extent of PPI including the availability of resources, purpose and relevance of PPI in the context of this project and PPI contributors’ availability and interest in the extent of involvement. These factors also contributed to the use of traditional types of involvement when considering the involvement of BAME-PPI contributors.

Overall involving PPI contributors has been extremely valuable and has led to a better and more meaningful project. Involving a PPI contributor when developing the proposal for the doctoral research project was invaluable as it validated the timely need to explore this specific topic and provided direction when developing the research question for the doctoral research proposal. Working with PPI contributors throughout the project allowed for identifying some problems (e.g. redefining INVOLVE’s definition) which otherwise would have been unanticipated and develop solutions to overcome these barriers, confirm and validate that right decision have been made and increasing researchers’ confidence in those decisions. AR and VP provided feedback regarding the recruitment of South Asian participants, which enabled the researcher to reconsider the approaches to advertising participation opportunities and use diverse approaches for participant recruitment. For example, information sheet and consent form were translated to Urdu and Gujarati by an external agency pre-vetted by the University as this was the commonly spoken language amongst South Asian community groups in the local area. Based on their suggestion, a poster was taken to advertise in a local Asian supermarket. This opportunity helped in identifying an individual who attended a local community group and was an active member of the community. They initially volunteered to participate in the study and then supported SD to gain access to the local community groups. As the community group members had an established relationship with this individual, it enabled the researcher to engage with them, which otherwise would not have been possible. Furthermore, PPI contributors advised the researcher to recruit a few participants and identify additional participants through them (chain referral sampling). Chain referral sampling was used as a complementary approach in addition to purposeful sampling, and it was immensely helpful as trust was already established amongst people belonging to the same community.

Involving PPI contributors had an impact on research quality and relevance. For example, both the PPI contributors also participated in developing and testing the interview schedule as participants and changes in the form of wording, ordering of questions with made to the interview schedule. Together we also refined INVOLVE’s definition of PPI and used some examples to help explain what PPI is to the lay participants. For example, we defined involvement as working with members of the research team and examples to explain ways to get involved included, feedback obtained in developing the patient-facing materials, interview schedule used to interview participants. This was important because most of the participants in the study were not aware of PPI opportunities and finding a way to explain this concept was important. The researcher and the PPI contributors were involved in a range of engagement activities during this doctoral research. This included advertising research participation opportunities, dissemination event, sharing their experiences of PPI via the NIHR GM PSTRC blog, Primary Care Research in Manchester Engagement Resource (PRIMER) at 10 event, plain English summary for Greater Manchester PSTRC website and Greater Manchester PSTRC dissemination event. A few lay participants since taking part in this study and those who attended the dissemination event have since become involved as PPI contributors in other doctoral research projects.

Involving two PPI contributors rather than setting up an advisory group facilitated the development of a good relationship with PPI contributors where they were ‘critical friends’ and encouraged an open and honest conversation. AR and VP had different sources of experiential knowledge (e.g., AR with some PPI experience and VP no previous PPI experience). While our meetings had some structure enabling targeted dialogues, it also facilitated sharing and mobilisation of experiential knowledge and enabled mutual learning and reflection. Given the context, sharing personal stories at meetings allowed for an amalgamation of different types of knowledge, which either complemented or fine-tuned previous knowledge. This harmonious combination of individual and collective knowledge and experience enriched the research project, facilitated learning. It filled gaps in the researcher’s experiential knowledge as it allowed for an examination of a variety of issues with careful consideration without making assumptions. For example, PPI contributors’ experiential knowledge allowed the researcher to consider alternative approaches to advertising participation opportunities (e.g. a local community supermarket) and navigate cultural barriers. Furthermore, it also enabled the researcher to take measures that are culturally appropriate to encourage and facilitate engagement from female participants of Pakistani origin for and during the dissemination event; for example, providing a separate women’s only table(s) with female interpreters. Without their insightful experiential knowledge, this opportunity would have been missed, and as a result, this group would have been unintentionally excluded from being engaged at the dissemination stage. Discussing study findings was stimulating as it allowed the researcher to learn about their life experiences and how some of the findings resonated with them. It also led the researcher to appreciate that while some of the issues were cultural others were broadly generalisable.

A range of values and principles to involvement was adopted when undertaking this doctoral research. This can be mapped onto the values and principles framework of good practice for PPI in research developed by INVOLVE [24]. Values include respect, support, transparency, responsiveness, fairness of opportunity, and accountability. Table 2 highlights some examples from the doctoral research mapping onto these values. These values were crucial as they facilitated involvement in this research and also supported the continued involvement of the PPI contributors, and led to developing and fostering a positive relationship. However, these values and principles exist, they are rarely used by researchers to explain involvement or good practice for PPI in research projects.

**Table 2:** Mapping doctoral research principles in practice against the values and principles framework

Values	Summary principles	Doctoral research principles in practice
**Respect**	Researchers, research organisations and the public respect one another’s roles and perspectives	• Different PPI contributors were involved in decisions about research in different ways from being involved at proposal development to dissemination of research. • PPI contributors were acknowledged for their contributions in the PhD thesis and they will co-author publications.
**Support**	Researchers, research organisations and the public have access to practical and organisational support to involve and be involved	• Doctoral researcher (SD) had the opportunity to attend various PPI training that supported PPI in this research. • SD provided educational session to PPI contributors on systematic reviews, qualitative research especially data collection and analysis. • Planning PPI and related activities from the outset enabled allocation of realistic timelines for PPI input and incorporating that into the different stages of the research project. • Allocated costs to undertake PPI throughout the doctoral research to cover for their time and expenses. • The Greater Manchester PSTRC provided infrastructure that supported PPI in their research and allocated 6% of its total budget to PPI.
**Transparency**	Researchers, research organisations and the public are clear and open about the aims and scope of involvement in the research	• Initial meeting to discuss ground rules, expectation management, clarity on roles. Discussion around levels of involvement including type of contribution and duration of involvement. • PPI contributors were open about their availability, time commitment and ability to contribute. • Payment policy was outlined from the outset so the contributors knew what type of payment they would receive, for the type of work they would receive and childcare and travel costs would be covered.
**Responsiveness**	Researchers and research organisations actively respond to the input of public members involved in research	• While we had an agenda for meetings, there was flexibility to voice opinions at any time point during the meeting and the agenda was to offer structure rather than a setting stone for what was discussed and how. • Feedback from PPI contributors were incorporated at different stages of the research project.
**Fairness of opportunity**	Researchers and research organisations ensure that public involvement in research is open to individuals and communities without discrimination	• PPI contributors from diverse backgrounds were involved at different stages of the research project. • Building trust and relationship with PPI contributors during the research project allowed to sustain long-term involvement and for the PPI contributors to understand the research and processes involved. • Arrangements were made to ensure that the venue was accessible, paper copies of the documents that required reviewing was posted to the PPI contributors. • New PPI contributor (VP) had the opportunity to choose whether to be involved in the wider Greater Manchester PSTRC RUG.
**Accountability**	Researchers, research organisations and the public are accountable are accountable for their involvement in research and to people affected by the research	• Keeping PPI contributors in loop from start to finish and between meetings to maintain regular contact regarding progress. • Study participants were invited to take part in the dissemination event. • PPI contributors were made aware of how their input had an impact on the project. • Learning and reflecting on PPI in this doctoral research project through this paper.

## Discussion

There is little evidence describing PPI in doctoral research [25, 26]. Moreover, researchers rarely provide a complete picture of what happened during the research process. To our knowledge, this paper is novel as we present the journey of the PhD research project and PPI with examples of how PPI was conducted during the doctoral research (pre-funding to dissemination), personal accounts of the PPI contributors and the doctoral researcher reflecting their experiences along with the impact of PPI. This paper is an exemplar of how PPI at different stages of doctoral research can be undertaken with examples illustrated, also echoing the need to embed PPI within doctoral research.

The results highlight that PPI improved and contributed to study recruitment and participation similar to the extant literature [27, 28]. PPI also had an impact on research quality and relevance (e.g. appropriateness of the interview schedule), the researcher and the contributors themselves. At different stages, the involvement of PPI contributors enacted as a catalyst to tackle problems by finding practical solutions reflecting the findings from the wider literature [29–31]. We have also outlined some practical approaches that were considered when involving PPI contributors at different stages with involvement being fluid, and no particular approach to involvement was used as a benchmark for gold-standard PPI. Using such an approach also highlights the benefits such involvement of PPI contributors can bring to different aspects of the research. For example, involving additional PPI contributors proved beneficial and added value when developing the systematic review protocol as it enabled the identification of new terms to the search strategy and the definition for BAME groups. Improving the considerations of the range of experiential expertise can potentially improve PPI planning and practice. For example, additional involvement of people with prior (and extensive) PPI experience was most relevant and of value, as it led to the identification of additional search terms which wouldn’t have been possible otherwise. This reflects that different kinds of experiential knowledge are relevant in different contexts. Working with various PPI contributors meant that diverse perspectives were taken into account when making decisions at different stages of the research process. Moreover, involvement in the review protocol provides a classic example that also helps address the fundamental questions about when, why, and whom to involve in a PPI capacity. Given the topic area of the doctoral research, involving PPI contributors with different experiential knowledge (varying levels of PPI experience) was appropriate as it benefitted the research project, filled gaps in the researcher’s experiential knowledge, and facilitated mutual learning. This suggests a need for *‘experiential representativeness,’* i.e., representation of people with the experiential knowledge that is most relevant to work being done [32]. During the meetings, we not only learnt about similar and different life experiences but being involved in the project also enabled PPI contributors to learn more about research and research process. Moreover, it is also essential to recognise the value of experiential knowledge provided by the PPI. In this doctoral research, we recognised their value through different means such as compensation for their time, an acknowledgement in all presentations, PhD thesis, dissemination at various events, and co-authorship in articles.

Time and financial constraints are common challenges to PPI [17, 31] with a mismatch between funding timelines and fieldwork time needed to engage with groups cited as an issue [33]. This was not the case in the present study as PPI contributors’ suggestions regarding linking with community gatekeepers/leaders as a means to engage and reach South Asian participants led SD to establish strong community networks prior to recruitment. This otherwise could have been time intensive and may have impeded recruitment. Additionally, this also contributed to the attendance of the dissemination event by the study participants and subsequent involvement in a PPI capacity by a few participants in other PhD research projects. Some of the examples provided in this paper are not only useful when considering PPI in doctoral research but can be translated to areas of engagement and participation to foster and promote inclusivity and diversity.

Planning PPI activities and allocation of NIHR advised funding from the outset and support from the supervisory team made this level of involvement possible. Considering and implementing PPI from the pre-funding stage and planning potential PPI activities meant that PPI was well embedded in this research with the research project delivered within the timeframes without compromising the quality of PPI. While we involved individual PPI contributors instead of setting up an advisory group, we acknowledge that there is no one-size-fits-all approach to involvement and that different approaches may be appropriate for different contexts [21, 34].

Building and developing rich relationships over time facilitated the involvement of PPI contributors throughout the 4 years and beyond. Our experiences on the need to take a flexible approach, clarifying expectations, need to build trust and relationships supports the findings identified in the wider literature [21]. Involving PPI contributors from the outset, offering them flexibility and understanding their expectations from involvement allowed to avoid tension over who has control of the research. Relationships built and the quality of the relationship also contributed to avoiding tensions allowing to rebalance power with the research process. Reciprocity in research practice [35] along with mutual respect and active learning [36] is a useful principle to consider fostering long-term involvement of PPI contributors and to enable building trust and relationship. Examples presented in this paper also highlights that not all involvement outcomes were the result of the researcher’s efforts but a collective effort by both the researcher and the PPI contributors. This in-turn helped facilitate meaningful involvement of PPI contributors and optimum level of involvement that met the needs of the research project.

Need for support and training for researchers and PPI contributors have been recognised as vital elements that should be promoted to facilitate involvement [37, 38]. SD attended various research methods and PPI related training and provided a variety of education sessions for AR and VP on systematic reviews, research methods, analysis and facilitation. This not only facilitated involvement but also encouraged the PPI contributors to engage in decision-making meaningfully. Training increased the researcher’s confidence to deliver PPI throughout the research project. It is important that doctoral candidates are aware of PPI, signposted to relevant training, resources available to them in order to plan and budget for PPI and receive supervisory support to be able to undertake PPI activities. In future, researchers should consider using available resources when planning to submit their pre-doctoral or doctoral or advanced fellowship applications. For example, when considering applying for an NIHR fellowship or grant, researchers should consider seeking support from their local Research Design Service for PPI related advice and support to cover involvement costs. Furthermore, many research organisations now have dedicated PPI co-ordinators or leads who can support researchers in identifying PPI contributors and support them in planning PPI activities as a part of their research application.

Funders such as the NIHR now expect researchers applying for pre-doctoral, Doctoral Fellowships, Advanced Fellowship and Development and Skills Enhancement Awards to show evidence of PPI being a part of the preparation of applications and plans to involve PPI contributors in proposed work. There are various training opportunities on PPI offered by NIHR^2^ and other organisations for researchers with different levels of knowledge of PPI across the UK. Moreover, NIHR annually organises a training camp for doctoral researchers funded by the Biomedical Research Centres, Collaborations for Leadership in Applied Health Research and Care, Patient Safety Translational Research Centres, Health Protection Research Units, the School for Primary Care Research, the School for Social Care Research and the School for Public Health Research. This training camp provides doctoral researchers with practical experience to learn about different aspects of research from applying for funding to involving PPI contributors and costing for PPI.

Based on our experience of undertaking this research project, we have proposed some recommendations when considering the involvement of PPI contributors which is relevant for doctoral and postdoctoral researchers. Table 3 presents some learnings and recommendations from undertaking PPI in this doctoral research project. This is crucial for ensuring that PPI within doctoral research can be done well with careful planning without it becoming time or resource-intensive or a tokenistic exercise.

**Table 3:** Recommendations from this research project

- Plan and allocate resources to undertake PPI - Identify training opportunities on PPI and offer PPI contributors training opportunities - Involve PPI contributors as early as possible - One-size does not fit all, so consider a combination of different approaches to involvement - Consider different experiential expertise which potential PPI contributors can bring to the table not just their experience of a condition but also other sources of experiences such as prior PPI experience, research participation experience as different experiential knowledge is relevant in different context. - Building trust and relationship key to maintaining relational dynamics can contribute to continued involvement - Building trust and links with community group leaders is needed to engage with BAME groups - Fostering a culture where PPI contributors as critical friends-a valued component of the research process - Impact of PPI at some stages can be as simple as validating decision-making or findings - Record all PPI activities during the course of the research cycle - Careful monitoring to ensure that intended involvement translates to practice	

In line with the reporting recommendations in Guidance for Reporting Involvement of Patients and the Public (GRIPP2) short form [39], we have reported on all aspects of the processes involved in undertaking PPI. There are some limitations that need to be acknowledged; while efforts were made to involve and engage PPI contributors from diverse backgrounds, traditional methods were used to involve them. This was because of time and resource constraints, and therefore no novel approaches to PPI were considered. No formal or informal evaluation was undertaken, and the informal training offered to the PPI contributors were delivered by the doctoral researcher and not an external trainer which may have led to some bias. While the qualitative studies in the doctoral research focused on both South Asian people’s involvement in PPI and researchers’ experiences, we did not involve researchers in this project. However, within the supervisory team, researcher (RM) had the experience of working with people from BAME groups using community-based participatory approaches.

## Conclusion

This work contributes to building evidence-based practice enabling researchers to focus on effective forms of involvement, which is scarce in the current literature. In this paper, we have presented an exemplar of PPI journey in doctoral research from how PPI was embedded in a doctoral research project, its overall impact, and personal accounts of PPI contributors and researchers involved. For PPI to be viewed as an integral part of the research process and increase its value to the research project and stakeholders involved, it is essential to normalise PPI as a part of the research process rather than an add-on feature. We have provided examples of the principles and values of PPI that allowed for building and maintaining long-term relationships, facilitated continuity of involvement along with recommendations that could help improve the opportunities for learning for potential doctoral and postdoctoral candidates when considering meaningful PPI in the future.

## Data Availability

Not applicable.

## Abbreviations

BAME: Black, Asian and Minority Ethnic
GRIPP: Guidance for Reporting Involvement of Patients and the Public
NIHR: National Institute for Health Research
PPI: Patient and Public Involvement
PRIMER: Primary care Research In Manchester Engagement Resource)
PSTRC: Patient Safety Translational Research Centre
RUG: Research User Group

## Footnotes

1 https://www.invo.org.uk/posttypepublication/involve-policy-on-payments-and-expenses-for-members-of-the-public-including-involve-group-members-february-2016/

2 https://www.spcr.nihr.ac.uk/PPI/resources-for-researchers/training-for-involvement-and-engagement
